# PW03-021 - HSCT in mevalonate kinase deficiency

**DOI:** 10.1186/1546-0096-11-S1-A247

**Published:** 2013-11-08

**Authors:** B Wolska-Kuśnierz, B Mikołuć, R Motkowski, K Kałwak, E Bernatowska, D Rowczenio

**Affiliations:** 1 Immunology, Children's Memorial Health Institute, Warsaw, Poland; 2 Pediatrics and Developmental Disorders of Children and Adolescents, Medical University, Białystok, Poland; 3 Pediatric Hematology and Oncology, Medical University, Wroclaw, Poland; 4 National Amyloidosis Centre, London, UK

## Introduction

Mevalonate kinase deficiency (MKD) has a wide spectrum and severity of clinical manifestation. Patients with mutations in MVK gene leading to complete lack of the enzyme, suffer from the most severe form of disease, also known as mevalonic acydosis, whereas defects with preserved, but insufficient enzyme activity present with autoinflammatory syndrome , also known as hiperIgD syndrome (HIDS). Both diagnosis and treatment of MKD is a great challenge for clinicians. Hematopoietic stem cell transplantation (HSCT) is the only available therapy that allows delivery to the tissues of the missing enzyme produced by healthy donor hematopoietic cells.

## Case report

We present a case of 4-year-old girl with genetically confirmed mevalonic kinase deficiency.

She has suffered from recurrent fever, lymphadenopathy, hepatosplenomegaly, hepatitis, impaired growth since she was born. Constant mevalonic acyduria was the first key for diagnosis, then confirmed by genetical analysis of MVK and lack of MK enzyme activity in blood cells. The response to treatment with steroids and IL1-blocker was poor, with only partial resolution of autoinflammation and steroid-dependent adverse events. Due to worsening clinical condition, at the age of 2.5 years patient received bone marrow transplantation (BMT) from matched sibling donor. Due to decreasing donor chimerism, the girl required second transplantation from the same donor at the age of 3 years, followed by repeated donor lymphocyte infusions (DLI). Five months after last DLI the patient achieved stable full donor chimerism. At last follow up, 12 months after HSCT, the girl is in excellent clinical condition, without signs of autoinflammation for 7 last months, progress in psychomotorical and physical development and good immunological reconstitution was observed. Activity of mevalonate kinase is present after transplantation, although still below normal range and mevalonic acyduria is still observed. To assess further improvement of laboratory markers and its clinical consequences longer follow up of the patient is required.

## Discussion

Presented case encourages to qualify patients with severe course of MKD to hematopoietic stem cells transplantation.

## Disclosure of interest

None declared

